# Metagenomic 16S rDNA amplicon data of microbial diversity of guts of fully fed tropical bed bugs, *Cimex hemipterus* (F.) (Hemiptera: Cimicidae)

**DOI:** 10.1016/j.dib.2020.105575

**Published:** 2020-04-18

**Authors:** Li Lim, Abdul Hafiz Ab Majid

**Affiliations:** Household & Structural Urban Entomology Laboratory, Vector Control Research Unit, School of Biological Sciences, Universiti Sains Malaysia, Minden, Penang 11800, Malaysia

**Keywords:** Cimex hemipterus, Metagenome, Microbial DNA, Proteobacteria

## Abstract

The metagenomic datasets of the microbial DNA from tropical bed bugs (*Cimex hemipterus*) after feeding on human blood were presented. Next-generation sequencing of the community DNA was carried out on an Illumina Miseq platform and the raw fastq files were analyzed using QIIME (version 1.9.1). The metagenome of three samples comprised of 108,198 sequences representing 44,646,263 bps with a mean length of 412.63 bps. The sequence data is accessible at the NCBI SRA under the bioproject number PRJNA600667. Community analysis showed Proteobacteria was the most abundance (more than 99%) microbial community that present in the guts of fully fed tropical bed bugs.

Specifications table

**Table:** 

Subject	Microbiology
Specific subject area	Metagenomic study on the microbial community in the guts of *Cimex hemipterus*
Type of data	Figures, table and 16S rDNA Illumina sequence
How data were acquired	16S v3-v4 amplicon metagenomics sequencing followed by community metagenome analysis.
Data format	Raw fastq files
Parameters for data collection	Laboratory strain tropical bed bugs after feeding on human blood
Description of data collection	The microbial DNA was extracted from the crushed guts of fully fed tropical bed bugs using HiYield™ Genomic DNA isolation kit (Real Biotech Corporation, Taiwan). 16S v3-v4 amplicon metagenomics sequencing was carried in Illumina MiSeq platform.
Data source location	Visual inspection was conducted with the aids of flashlight and the bed bugs were collected using forceps from cushion seat at the waiting area in Kuala Lumpur International Airport (KLIA) (25 Feb 2014) at the coordinates of 2.7456 N 101.7072 E.
Data accessibility	Repository name: NCBI SRA
	Data identification number: PRJNA600667
	Direct URL to data:
	https://www.ncbi.nlm.nih.gov/bioproject/PRJNA600667

## Value of the data

• The metagenomics data provide full taxonomic profiles of the microbial diversity and abundance in the guts of fully fed *Cimex hemipterus*.
• The data also provides an initial picture of the functional capabilities of the gut microbial community of *C. hemipterus*.
• The data is important for profiling, annotation or pathway reconstruction in understanding the metabolic process within the guts of *C. hemipterus* performed by microorganisms for forensic and research purposes
• Provide information regarding the microbial community that may associate in blood meal digestion or pathogen defense of *C. hemipterus*
• Provide possibility in recovery of novel biocatalysts from metagenomic data
• Provide information on the bacterial species and functional groups that play important role for the host's success, offers scope for future studies to develop new pest management approaches that exploit novel targets of chemical, genetic and biological control for *C. hemipterus* and other insect pests.

## 1 Data description

The Illumina Miseq sequencer produce 32,816, 43,211, 32,171 sequences with average read length of 413.50, 411.61, 413.13 from samples BB1, BB2 and BB3 respectively (Table 1).

**Table 1:** Number of sequences, base pairs and average length of the sequences from each sample.

Sample	Sequences	Bases (bp)	Average Length (bp)
BB1	32,816	13,569,406	413.50
BB2	43,211	17,786,156	411.61
BB3	32,171	13,290,701	413.13

**Fig. 1:**
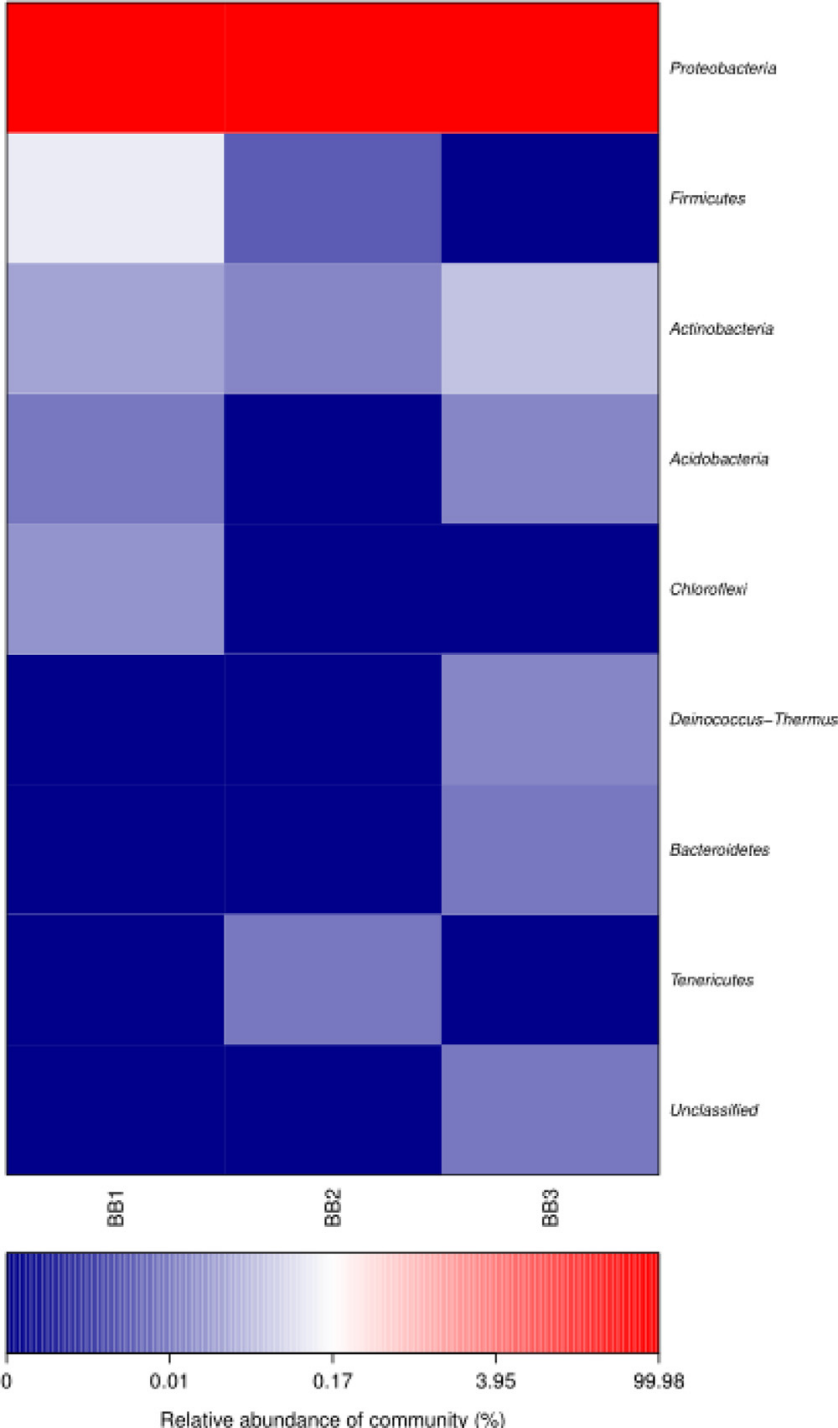
Heatmap analysis which showed the relative abundances of microbial community in *C. hemipterus* individuals (Samples BB1, BB2 & BB3).

The community analysis showed that more than 99% of sequences of the three samples were assigned to two families within the Proteobacteria: Anaplasmataceae and the Enterobacteriaceae. Two genera including *Wolbachia* and *Pectobacterium* comprised the majority of these families. Less than 1% of the reads from 7 phyla of bacteria including Firmicutes, Actinobacteria, Acidobacteria, Chloroflexi, Deinococcus-Thermus, Bacteroidetes, Tenericutes and unclassified reads (Fig. 1).

## 2 Experimental design, material and methods

**Fig. 2:**
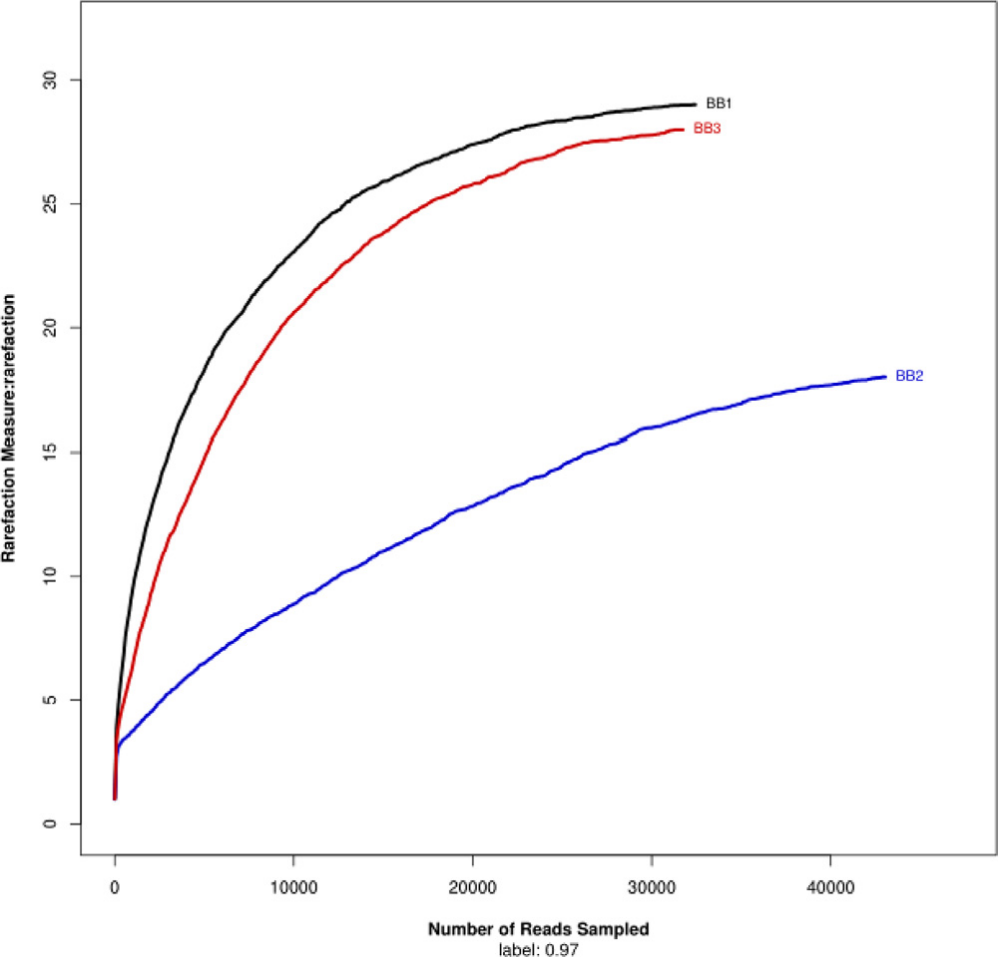
Rarefaction curve showing the species richness of the microbiome of *C. hemipterus* individuals. Samples BB1 (black curve), BB2 (blue curve) and BB3 (red curve) (For interpretation of the references to color in this figure legend, the reader is referred to the web version of this article.).

The microbial DNA was extracted from guts of fully fed tropical bed bugs (*C. hemipterus*) with three replications (BB1, BB2 and BB3) using HiYield™ Genomic DNA isolation kit (Real Biotech Corporation, Taiwan) following the manufacturer's protocol. 16S v3-v4 amplicon metagenomics sequencing was completed via the Illumina MiSeq platform according to the standard protocol. The sequences were analysed using QIIME (version 1.9.1) [1]. Low quality reads with average quality score <20 were trimmed with Trimmomatic [2] software and trimmed reads with lengths shorter than 50 bp were discarded. Paired-reads were merged became single read using FLASH (Fast Length Adjustment of Short reads) [3] based on overlapped relationship. Sequences that overlap longer than 10 bps were assembled while reads that could not be assembled were discarded. The merged reads were used to Operational Taxonomic Unit (OTU) clustering with 97% similarity cut-off using UPARSE [4] software while chimeric sequences were detected using UCHIME [5] software and were removed from the analyses. The taxonomy of the 16S rRNA gene sequences were analyzed using RDP Classifier [6] against the SILVA 16S rRNA database [7] with confidence threshold of 0.7. The sequence coverage of each sample (BB1, BB2 and BB3) was evaluated by rarefaction analysis (Fig. 2) using mothur [8] and R [9] software. The detailed protocol was available on dx.doi.org/10.17504/protocols.io.bc9giz3w.
